# Selections and Abstracts

**Published:** 1915-03

**Authors:** 


### SELECTIONS AND ABSTRACTS

### THE INTRAVENOUS USE OF COLLOIDAL (GELATIN) SOLUTIONS IN SHOCK.

JAMES J. HOGAN, M. D., M. R. C. S., ENG. SAN FRANCISCO.

### I INTRODUCTION.

Periodically in medical and surgical practice we are brought face to face with a series of signs and symptoms that are in essence the expression of an abnormally low blood pressure, and which, when sufficiently marked, serve to kill a patient.

Not alone is the list of pathologic states in which a low blood pressure is observed very long, but the explanations given by different authors as to why the low blood pressure exists in any given state are very different. From a therapeutic point of view, however. this ground is held in common by all authors: They are agreed that a saving of life is intimately associated with, or rendered possible only by our success in raising the blood pressure, and keeping it up until such time as the patient himself succeeds in overcoming the conditions which are responsible for the low blood pressure.

Simple as it would seem to attain this end, its accomplishment practically is still a matter of debate. The problem involved is perhaps best presented if we consider the effects of simple hemorrhage. If by accident or otherwise, one of the larger vessels in man or a laboratory animal is opened, we observe following each other in rapid succession and in the course of a very few minutes all those alarming signs which reach their climax in death.

When now we try to say why this occurs, it is quickly brought home to us that the most serious mischief done by the hemorrhage does not reside in a great loss of red blood corpuscles or in a loss of certain of the chemical constituents found in the blood, say the hemoglobin or certain salts, but in a diminution in the volume of the circulating blood. The proof for such a conclusion is easily brought, for to protect or save an animal from the effects of hemorrhage it is not necessary to transfuse blood; but transfusion with water containing various salts (so-called "physiologic salt solution," "Ringer solution" or "Locke solution") may do. In fact, the dangers incident to transfusion of whole blood have made medical men in practice depend more and more on salt solutions of various kinds and less on the transfusion of blood itself.

But even though salt solutions of various kinds work excellently, they do this for only a limited time. In other words, it is too often noted that while physiologic salt solution or a Ringer solution produces immediately brilliant results, this effect wears off in an hour or two, so that the individual who has been roused from the threatening effects of a great hemorrhage begins to sink again, and even though we repeat our injection, the improvement in patient or animal is again only temporary.

When we ask why this occurs we have no difficulty in finding the answer. The injected salt solution does not remain in the blood vessels. The evidence for this is perfectly clear. Not only does the blood pressure, which we have brought up after the hemorrhage, slowly fall again, but the fluid that has been injected appears as urine, or it is taken up by the tissues (an edema develops), or both. Clearly, if the water could be kept in the blood vessels, the temporary effects following the injection of salt solutions could be made more permanent.

This brings us to the heart of the problem. Why is a salt solution not retained in the blood vessels of an animal, why does a circulating liquid like blood remain in the blood vessels? Why does blood not suffer the same fate as a salt solution and be lost from an animal as urine or be absorbed into the animal's tissues?

As is self evident, a correct answer to this question is not only of general biologic interest, but also constitutes the basis on which must be built the principles underlying the whole problem of perfusion as practiced for any cause whatsoever in a clinical case.

The blood (or the lymph) remains in the blood vessels because it contains no "free" water—all the water in it is held (as hydration water) in combination with the colloids present here. A salt solution rapidly disappears from the vascular system for the reverse reason, in other words, because this does contain "free" water.

The detailed experimental proof for the correctness of this reasoning was brought some time ago. I review here a few experiments which illustrate our contentions, after which I shall discuss the results which have been obtained in human cases in utilizing the principles learned on animals.

### II. EXPERIMENTAL DATA.

1. If we take a rabbit quietly from its cage, tie it comfortably into an animal holder and catheterize it with a small soft rubber catheter, we get a urinary secretion that is represented graphically in the lower-most curve (a) of Chart 1. This, as all the other curves, is obtained by plotting time along the horizontal (in fifteen-minute intervals), and the number of cubic centimeters of urine secreted in each of these intervals on the vertical.

Let us now see what is the effect on this secretion of injecting intravenously at a uniform rate (10 c.c. every five minutes) an amount of differently concentrated sodium chlorid solutions (about 125 c.c.) which equals in volume the circulating blood in the animal. The result is indicated in the remaining curves.

In Curve b is shown the effect of injecting a one-eighth molecular (0.729 per cent.) sodium chlorid solution. As is readily apparent, the injection of a weak salt solution is quickly followed by an increase in the urinary output.

Curve c shows the effect of injecting the same volume of a salt solution that is more nearly "isosmotic" or "isotonic" with the tissue fluids of the rabbit—namely, a 0.92 per cent, sodium chlorid solution. We have no difficulty in seeing that the urinary output is under such circumstances still further increased. Also we notice that the effect of this solution on the secretion occurs earlier, even though the quantity of solution injected and the rate of injection are the same as in the previously described experiment.

Curves d and e show the effect, respectively, of injecting one-fourth molecular (1.459 per cent) and one-half molecular (2.909 per cent) sodium chlorid solutions intravenously. Evidently, with the injection of a constant volume of water at a constant rate, the loss of water from the body occurs the more rapidly, the higher the concentration of salt injected along with the water. Little or none of the water that is injected is retained, and if enough salt is injected with the water, even more water is lost by the animal than is injected.

Let us turn now to the interpretation of these simple experimental facts.

The whole animal, that is, all his tissues, including the blood and lymph, is composed of a system of colloids which under normal circumstances are saturated with water. This system of colloids cannot take up any water or liberate any, except as it first suffers changes of a chemical or physicochemical character that either increase or decrease the capacity of these colloids for water. If the system of colloids composing the body is saturated with water, then clearly it cannot take up any more of this substance, and so if we inject "free" water intravenously in the form of a "physiologic" sodium chlorid solution, this cannot be retained in the body. It escapes as urine. If, instead of doing this in an experimental animal, we have employed this procedure in a patient suffering from hemorrhage, the result and the interpretation of the result are just the same. The good effects on the patient resulting from filling up his blood vessels with salt solution can be only temporary, for the water is "free" and escapes shortly after the injection as urine.

But why does a strong salt solution make the water come out as urine sooner than a weaker solution, and why in a greater amount?

It has generally been held that this is because the salt "stimulates" the kidney cells in some mysterious way. Aside from the fact that this means nothing, the explanation is really simpler. The salt decreases the capacity of the body colloids (the protein colloids) for holding water, and this the more, the higher the concentration of the salt. The higher the concentration of the salt in our solution, the higher must this therefore be in the blood, and secondarily (after diffusion) in all the tissues of the body. In injecting a strong salt solution we therefore not only supply free water as before, but by increasing the salt concentration in the blood and tissues, we make the colloids here give up water. This "free" water is then added to that already "free" in our injection mixture, and so the urinary output is increased pari passu with every increase in the concentration of the salt in the injected fluid.

2. We can at once test the soundness of our reasoning by substituting for the plain salt solutions one in which the solvent (water) is not free, but combined with a colloid.

The natural fluid that has these characteristics is of course, if our reasoning is correct, the blood, and so we should expect that the injection of no amount of blood would yield any increase in urinary output. That it does not is a familiar fact in physiology since the experiments of E. Ponfick and R. Magnus, only the interpretation of this observation has been lacking.

Chart p shows how the injection of a solution in which the water is combined with a colloid does not yield any increase in urinary secretion (it remains in the blood vessels). In these experiments we did not use whole blood, but sterile blood serum. In spite of the fact that the same amount is injected as in the experiments with salt solution, the curves showing the urinary output are identical with those which are obtained from normal animals which have already simply been comfortably tied into an animal holder.

3. It might now be argued that blood has something specific about it that makes it remain in the blood vessels, and that it does not simply because the water in it is held in combination with the blood colloids. This criticism is met in Chart 3, which shows the results of experiments in which water is again injected intravenously, only this time in combination with a colloid foreign to the blood, namely, gelatin. As the two curves show, a 1.5 to 2.5 per cent gelatin solution, when injected intravenously, remains in the blood vessels and does not yield any increased urinary output.

4. If the foregoing experimental facts are correctly interpreted, it is of course to be expected that we can at will make an animal retain water in its blood vessels or give this up, depending on whether the water so introduced is given in combination with a colloid or "free." This may be proved in one and the same animal. The results of injecting water in combination with a colloid as blood serum or a gelatin solution, and "free" water as in a salt solution, are shown in Charts 4 and 5. While no increase in urinary output is observable in the first period of these experiments in which blood serum or gelatin is injected intravenously, the urinary secretion rises as soon as a salt solution is substituted for the serum. The curves are clearly the syntheses of the results obtained before in separate animals when we injected colloidal solutions or salt solutions alone.

III. CRITICAL REMARKS.

To those who are disinclined to accept the teaching that the living animal represents simply a series of colloidal phases that are saturated with water, and that secretion becomes possible (in the main) only as some of this water is freed (as by increasing the salt concentration in the protoplasm of the animals), or "free" water is injected from without, these experiments, in which we take a secretion of urine or its absence as evidence that an injected fluid has retained in the blood vessels or not, may not be entirely convincing. We must meet their objections.

Mere examination of the animals while they are being injected with a colloidal solution or with a salt solution indicates that the fluid remains in the blood vessels in the former case and escapes from these in the latter. Inspection of the more superficial blood vessels (veins and arteries of the skin and ears, the carotids and the femorals) shows that, when a colloidal solution is injected, these become progressively fuller and remain so; as the vessels become more and more filled, the amplitude of the pulse waves becomes smaller and this effect persists. Even when we inject such a non-poisonous fluid as blood serum we can only inject a limited volume before we notice that the respiration of the animal is becoming embarrassed, and if we persist, the animal dies from mere over-filling of the blood vessels, and the mechanical hindrance that this offers to the maintenance of a circulation of the blood.

Intravenous injection of a salt solution is not followed by such consequences. Here the filling of the blood vessels is not so apparent, and the effect is not lasting. The respiration does not become embarrassed, and if we have previously overfilled the blood vessels with a colloidal solution and so induced a dyspnea, this is likely to improve if a strong salt solution is injected.

Further, if we have produced a (presumably) fatal hemorrhage and then saved our animal by the injection of blood serum, we can kill it subsequently by giving strong salt solutions which again decrease the volume of blood in the blood vessels.

That only the colloidal solution remains in the blood vessels is further brought home to us when we examine the injected animal at necropsy. In an animal that has been injected with a salt solution, especially if it has been a strong salt solution, the heart is normally distended with blood, or may appear underfilled, as do all the larger blood vessels. If the organs (liver, kidney, etc.) are cut across, they bleed a little or may be quite dry. If, on the other hand, a colloidal solution has been injected, the heart and large vessels are distended with blood, and when one of the parenchymatous organs is cut across, the blood fairly wells out of it.

We know that the colloidal solution remains in the blood vessels from yet other facts. It simply cannot get out. Its loss from the blood vessels is entirely analogous to the absorption of such fluids from any of the serious cavities or the gastro-intestinal tract. Water injected into the peritoneal cavity in combination with a colloid (blood, lymph, ascitic fluid, gelatin solution, agar-agar, native egg white, etc.) cannot be absorbed until it has been separated from the colloid, or the colloid has been removed (digested). The same is true for the absorption of the water out of these solutions when introduced into any other serous cavity or into the lumen of the intestine.

Finally—what will seem most convincing to many physiologists and clinicians—direct blood pressure measurements show that salt solutions do not remain in the blood, while water in combination with a hydrophilic colloid does. As is known to everyone, the rise in blood pressure which follows the intravenous injection of a 0.9 per cent sodium chlorid solution into a normal rabbit is only temporary. In a few minutes to half an hour, the pressure has dropped back to normal. Accompanying it we have an increased urinary output. But if the same amount, say the equivalent of the calculated volume of blood in the animal, is injected in the form of blood serum or gelatin solution, the rise in arterial blood pressure (amounting to from 10 to 25 mm. of mercury) remains for hours; and in this case no increase in urinary output is noted. Especially marked are these findings if we first induce a state of low blood pressure in our animals by hemorrhage. While salt solutions give a temporary rise in blood pressure (and improvement in the general symptoms consequent on the hemorrhage), we obtain a permanent rise only if we inject a (hydrophilic) collodal solution.

IV. PRINCIPLES OF PERFUSION IN HUMAN CASES.

The foregoing experiments were made in order to formulate more clearly the principles that must guide us in combatting the various pathologic conditions encountered clinically which are characterized by an abnormally low blood pressure of such grade that it in itself threatens life.

Such a low blood pressure may result from any one or any combination of the following causes: (1) a decrease in the force or the number of the heart beats; (2) a diminution in the volume of the circulating blood; (3) an increase in the capacity of the blood vessels to hold blood. Or to illustrate this in ordinary clinical terms, a low blood pressure may result from a weakened heart muscle (myocarditis); from a hemorrhage, or a loss of the watery constituents of the circulating blood (oligemia); from a vasodilatation due either to a loss of tone in the blood vessel walls themselves or to impairment of the nervous (so-called vasomotor exhaustion) or chemical mechanisms (loss of the active principle of the suprarenal bodies from the blood) which are in part or wholly responsible for the maintenance of this tone.

In this paper I shall disregard the question of failure of the heart itself as responsible for a pathologically low blood pressure, and discuss merely the principles involved in our ordinary therapeutic attempts at restoring a low blood pressure to normal by introducing fluids intravenously.

It is clear from what was said above that only a proper colloidal solution can be injected intravenously with the hope that it will remain in the blood vessels and so maintain any rise in blood pressure gained by such means over any considerable period of time. But what colloidal solution may we or is it best to inject?

After what has been said it must be self evident that the best transfusion fluid is whole blood. But the obvious difficulties and dangers attendant on the carrying out of a man-to-man blood transfusion limits it usefulness. The liquid of next choice for perfusion is that which most nearly approaches blood, namely, blood serum. Human blood serum is, however, difficult to obtain. All the possibilities of obtaining a human colloidal solution that will stay in the blood vessels resides in the use of hydrocele fluid and of the ascitic accumulations from patients with heart disease. Such fluids can be drawn when opportunity offers into sterile containers and the serum separated from the fibrin clot by the methods employed in collecting horse serum. The use of the serum from human milk is also to be counted in here. Certain dangers are, of course, attendant on the use of any of this human material, but on these not too much emphasis is to be laid, for perfusion is not at present employed for patients who are not desperately ill.

It was to meet these various objections that a colloidal solution suitable for intravenous injection in clinical cases and obtainable from other than human sources was sought. The materials available for such use are limited, but as our previous experiments on animals and the clinical abstracts tend to show, properly prepared gelatin solutions may be used intravenously without danger.

PREPARATION OF GELATIN SOLUTION FOR INTRAVENOUS USE.

Twenty-five gm. of the purest gelatin, 1.5 gm. of sodium chlorid and 100 c.c. of distilled water are placed in a flask and boiled for fifteen minutes. The resulting solution is filtered through heavy paper in a hot funnel and then autoclaved for an hour at 124 C. (255.2 F.). It is then chilled in an ice-box. The gelatin has the proper colloidal qualifications if under these circumstances it solidifies. In this form the gelatin is kept until needed. In order to prepare the transfusion mixture, the flask is warmed until it melts, when it is added to 1,000 c.c. 0.9 per cent sodium chlorid to which have been added 2 gm. of sodium carbonate crystals (Na CO 10 HO) all warmed to body temperature.

The instructions as here given must be carefully followed, otherwise trouble will be encountered from the fact that, as ordinarily done, the heating necessary to steriilze properly the gelatin decomposes it and so destroys the very properties for which it is used. When heated in the presence of a sufficient concentration of salt, this decomposition is practically avoided.

The results that have been obtained by the use of such gelatin solutions intravenously are best illustrated by a few clinical histories. I am indebted to a number of my colleagues for permission to see their patients, and wish here to express my acknowledgements. Most of all I am indebted to Dr. B. F. Alden, who was the first to try the gelatin solutions intravenously in a series of injury cases in which death seemed the only prospect.

Case 1. (Dr B. F. Alden, San Francisco).—A. F., aged 39, Italian laborer, had always been in good health. March 3, 1913, he entered the French Hospital, the victim of a severe accident which had occurred the day before. A tree limb had fallen across his leg from a great height, crushing the upper end of the right tibia, and the head and lower third of the shaft of the femur. All structures beneath the skin, with the exception of the semitendonous tendon had been completely severed. There existed also a compound communicated fracture of the right tibia, fibula and ankle bones, which, however, did not bleed as all vascular communication had been severed above. The left tibia and fibula were fractured. Hemorrhage had been excessive, as practically no attempt had been made to control it. The man had been brought from the country and did not get into the hospital until twenty-four hours after the injury. The patient was in a state of profound shock. He was almost insanguinated and showed a small, rapid, soft radial pulse, with cold extremities, excessive thirst, and shallow and rapid respiration. Blood was oozing from the lacerated wound about the knee. Strychnin, normal salt solution subcutaneously gelatin subcutaneously, and the usual hemostatic measures were all used without improving the general state of the patient. Under ½ grain tropacocain anesthesia, administered intraspinally, the integument—which, with a single tendon alone united the crushed leg to the patient—was severed. The clotted blood and crushed bones were removed, and the large vessels in the stump were rapidly ligated; the wound was dressed and the patient rushed to bed, where his head was placed low and surface heat was applied. The pulse could now no longer be obtained at the wrist and, as death seemed imminent, it was felt that an intravenous injection of a gelatin solution was justified. Five hundred c.c. were given. As there was no radial pulse at the beginning of the injection, an accurate blood pressure reading could not be made. The needle of a Tycos instrument oscillated at 90 mm. While the injection was being made, the radial pulse gradually returned, and an hour later the blood pressure measured 125 mm. During the same time the pulse rate dropped from 130 to 88. The pressure was maintained, rising slightly from day to day, until on the tenth day after the infusion it measured 145, while at the same time the pulse gradually dropped to 78. The pressure gradually declined to 125 mm. and the pulse to 72. The patient made an uneventful recovery.

Case 2. (Dr. D. N. Richards, San Francisco).—Mrs. E. S. R. entered St. Francis Hospital, July 2, 1913, at 5:30 p. m., at full term and in labor. Regular pains continued until 4:15 a. m., when ether was given and she was delivered of a female child. The placenta was expelled at 5:10. This was followed by profuse bleeding, which continued until the patient was practically exsanguinated. At 6 a. m. the radial pulse could scarcely be felt. The pulse was 140, the blood pressure (verified by Drs. Richards and Fraser) 58. As the patient's condition was considered alarming, an intravenous injection of 750 c.c. of gelatin solution was given. The blood pressure rose to 118 mm. within an hour, the pulse fell to 100, and the patient's general condition improved greatly. For the following four days the pressure varied between 105 and 110 mm., the pulse between 85 and 100. The patient made a good recovery, leaving the hospital July 17.

Case 3. (Dr. B. F. Alden, San Francisco).—J. P. G., Frenchman, was operated on by an associate, February 7, 1913, for a ruptured appendix. A suppurative peritonitis with two fecal fistulas, one into the cecum, the other into a loop of the ileum, resulted. April 15, the openings in the intestine were repaired with Connell's interrupted sutures of silk covered by continuous Lembert catgut sutures. No excessive bleeding was noticed at the time of the operation, but one of the Connell sutures must have lacerated a large vessel, from which an active hemorrhage into the lumen of the intestine resulted. April 16, a lowered blood pressure with symptoms indicative of hemorrhage were observed. The outer wound was opened and the intestinal site explored, but as no serious bleeding was found, the intestine was not disturbed. The wound was packed with gauze. On the morning of April 18, a large bowel movement of coagulated blood was observed. In the meantime the patient had declined steadily. Ten c.c. of horse serum were injected subcutaneously. A second blood stool containing a large amount of fresher blood occurred, when another 10 c.c. of horse serum and 300 c.c. of physologic salt solution were given. The patient continued to decline, exhibiting all the alarming symptoms of active hemorrhage. At 3:30 p. m., April 18, the patient was reported dying. He was pulseless at the wrist. Oscillations in a Tycos instrument were observed at 80 mm. An immediate intravenous injection of 500 c.c. of gelatin solution was ordered. Half an hour later the patient's pulse had dropped from 156 beats to 138 and had gained in volume, strength and regularity. The blood pressure rose to 110 mm., and by the following morning it was 140 mm. This pressure persisted, while the pulse rate within three days gradually fell to 74. An uninterrupted convalescence followed, and at the present time this patient is well.

CASE 4. (Drs. W. B. Coffey and C. A. Walker, San Francisco).—Mr. B. entered St. Francis Hospital, July 26, 1913, having been ill for four days. The patient showed marked evidences of general intoxication. Examination revealed tenderness in the region of the appendix. An immediate operation was performed which revealed a ruptured appendix with marked circumscribed peritonitis. The patient showed marked shock following the operation. A 0.9 per cent sodium chlorid solution was dripped into the rectum throughout the day following the operation. The patient did not rally. The pulse became progressively weaker and rose to 130, while the blood pressure fell until at 9:30 p. m. it measured 86 mm. As death seemed the only outcome, gelatin solution was given intravenously. This was started at 9:50, the blood pressure at this time measuring 85 mm., the pulse 128. The following records show the rise in blood pressure during the injection:

After 150 c.c. it was 96.
After 250 c.c. it was 110.
After 400 c.c. it was 128.
After 500 c.c. it was 132.

As this pressure was deemed sufficiently high, the injection was stopped. The patient's general condition improved at once, the radial pulse filling out and the rate dropping to 90. The following morning the blood pressure was 119, the pulse 90. That afternoon the pressure rose to 128, while the pulse dropped to 85. The pressure remained, while the pulse gradually returned to normal. The patient made an uninterrupted recovery.

Case 5. (Dr. B. F. Alden, San Francisco).—M. C., aged 36, a native of France, because of an empyema of the left thorax following a lobar pneumonia, was transferred to the surgical service from the medical side, May 20, 1913, for a thoracotomy. Before he was brought to the operating room the patient showed the weak, thready pulse, rapid respiration, extreme pallor, drawn face, and cold and clammy extremities, ears and neck of profound shock. When placed on the operating table, the patient seemed in extremis, and was practically pulseless. Because of this, no proper blood pressure reading could be made. The anesthetist advised against the use of an anesthetic. As it was generally agreed that the patient would die, 500 c.c. of the gelatin solution were injected into the median basilic vein. The incision was made without anesthetic, as the patient was unconscious. Within ten minutes after the beginning of the perfusion, a perceptible radial pulse was noted, and this gradually improved in quality. A section of the eighth rib was now removed, and drainage of the pleural cavity effected. The patient recovered consciousness while on the operating table, and the blood pressure rose to 118 mm. It continued to mount until it reached 154 the following day. At the same time the pulse rate fell from 135 to 106. Two days later the pressure sank to 106, while the pulse mounted to 136. As the drainage was found to be inefficient, by reason of adhesions in the pleura, the patient was reoperated on under nitrous oxygen anesthesia. This was done without difficulty. The pressure then again rose to 126 mm. and remained there, while the pulse fell back to 100. At the present writing this patient has so far recovered as to be up and about.

Case 6. (Dr. B. F. Alden, San Francisco).—M. B., aged 54, a Frenchman, for some time preceding his operation had been unable to take food owing to an almost complete stenosis of the pylorus secondary to a carcinoma. He refused operation until May 13, 1913, when his condition had grown so grave from starvation that operative interference hardly seemed advisable. The blood pressure was less than 95 mm. As a prophylactic measure he was given 500 c.c. of the gelatin solution intravenously. The pressure rose rapidly to 115 mm., and it was decided to attempt an exploratory incision. An inoperable pyloric carcinoma being found, a posterior gastrojejunostomy was performed. Throughout the operation and afterward the patient's pulse and pressure continued good. After the operation he improved rapidly, vomiting but once several days later. In the afternoon after his operation the blood pressure was 120, on the next day 136 and for four days more 150 to 160, after which it fell gradually to 128, where it remained. The pulse rate fell in this same period from 95 to 86, and then more gradually to 76. At present he is walking about in better health than for months past. This improvement is due, of course, to his ability to take food, but the surgeons concerned felt that the necessary operation was made possible only because the patient was first perfused with gelatin.

It can, of course, be foreseen that what may actually be accomplished through gelatin transfusion in any case of low blood pressure depends on the intensity, persistence and nature of the factors responsible for it. Viewed in this light, it is not remarkable that our experience thus far has seemed to indicate that cases of shock consequent on simple hemorrhage are most easily relievable. Even those of our patient who showed extreme degrees of anemia rallied. Shock consequent on injury or a surgical operation in the ordinary "clean" case seems to be controllable almost as easily. Least hopeful are the septic cases in which the low blood pressure is in large measure dependent on a heart suffering from the general intoxication. This is illustrated in Case 7:

Case 7. (Drs. W. B. Coffey and C. A. Walker, San Francisco).—Mr. C., aged 32, a laborer, was brought into St. Francis Hospital at 1 p. m., July 25, 1913, with the diagnosis of a ruptured typhoid ulcer. He had had typhoid since July 1. It was felt that the patient could not live through an operation. The blood pressure was 65 mm., and as it was felt that the patient would certainly die, an intravenous infusion of gelatin solution was started at 2 p. m. After 300 c.c. had been injected, the pressure rose to 90 mm., after 400 c.c. to 96 mm., and after 500 to 99 mm. At this point the abdomen was opened under local anesthesia and found filled with fluid, fecal in character. The opening in the small intestine was quickly located and closed. The gelatin perfusion was continued during the operation, the blood pressure rising steadily even during operation as follows: after 600 c.c. had been injected it was 106 mm., after 700 it was 108 mm., after 850 it was 108 mm. This high pressure continued until 6:10 p. m., when he showed signs of a rapidly falling pressure and then died.

Besides this patient, I have seen two others with toxemic shock who died. One was a woman suffering from a hemolytic streptococcus infection of the blood stream after a miscarriage; the other, an ileus in the course of a peritonitis secondary to a ruptured appendix. In the former we raised the blood pressure from 75 mm. to 118 mm., where it remained for two days, when the patient died. In the second the pressure was raised from 58 to 124 mm. This patient also died.

As this paper deals only with the use of colloidal perfusion mixtures, I purpose discussing in a subsequent paper some additional methods at present available in the treatment of toxemic shock.—Journal A. M. A.

---

### ULCER AND CANCER OF THE STOMACH.
### THE CAMPAIGN AGAINST CANCER.

### Symposium: Looking to the Increased Knowledge Of Cancer.

### THE PRESENT STATUS OF CANCER RESEARCH.

By Leo Loeb, M. D., Philadelphia.

In the following brief discussion of the present status of cancer research, I shall have to limit myself to a mere outline of a few of the salient features, the necessary limitation of time precluding any attempt at completeness.

It is hardly ten years since we entered the experimental period of cancer investigation. We are in the beginning of our work and therefore not yet in a position to give definite answers to the most commonly asked questions.

Whether or not cancer is a parasitic disease can neither be affirmed nor absolutely denied at the present time, but I believe that on the whole the increase in our knowledge in recent years has not tended to the direction of the parasitic hypothesis. All attempts actually to demonstrate the presence of a microorganism have failed so far. But certain indirect evidence exists. In the first place, in restricted parts of certain towns, in certain villages or even in certain houses, the relative number of cancer cases was found much increased. The value of this apparent evidence in favor of infection is markedly diminished by the fact that the character of the tumor in these cases varied and that in certain cases hereditary influences could not be excluded.

Much more noteworthy are cases of the so-called endemic occurrence of cancer in animals. Hanau observed among a family of white rats three successive cases of squamous-cell carcinoma of the vulva, and in another family of white rats I found three cases of cystic sarcoma of the thyroid in the course of three years. If we consider that in the thousands of white rats which have been observed in various laboratories in this country, only one other case of cystic sarcoma of the thyroid can be found, the possibility that we have to deal with mere coincidences becomes exceedingly small. There exist, however, strong reasons for attributing these and other similar occurrences to hereditary conditions rather than to infection. But only systematic breeding experiments in animals, such as those carried out at the present time by E. E. Tyzzer, can definitely decide what part hereditary factors play in the pathogenesis of cancer.

The discovery that, in animals inoculated with carcinoma, sarcoma may grow besides the carcinoma might also be cited in favor of microorganisms as the cause of cancer. It suggests the transmission of microorganisms from epithelial to connective tissue cells, as a result of which the latter assume a sarcomatus growth. But other interpretations are possible. We can not absolutely exclude even a direct transformation of the the carcinomatous into the sarcoma cells. More recently, however, Carl Lewin observed, after the transplantation of a glandular carcinoma, not only the development of a sarcoma, but, at places of contact with the overlying skin, the carcinoma seemed to induce the proliferation of the epidermis leading to the production of a squamous-cell carcinoma, and again suggesting the transmission of a microorganism to the epidermal cells. But inasmuch as we have here to deal with an isolated observation it may be advisable to reserve judgment at the present time. If, then, no fact known so far necessitates the assumption of microorganisms as the cause of cancer, nevertheless their presence as intracellular parasites would explain very well the apparently endless proliferation of the cancerous cells. Without the hypothesis of the presence of such a constant, living stimulus, we will have to assume that the increased rate of cell-division, combined with an increased power of penetrating surrounding tissues and of metastasizing which characterize cancer cells, can be transmitted evidently to the following cell generations. In other words, we would have to assume a lasting inheritance of acquired characters in somatic cells. That such may be possible can not be denied, and perhaps it will be found to be the result of long-continued irritation. But at the present time it has not yet been definitely proved.

There can, however, be no doubt whatever that various nonspecific physical or chemical stimuli are among the best-established factors in the pathogenesis of cancer. Ordinary somatic cells, without any predisposing embryonal maldevelopment or without any postfetal misplacement, can in certain cases become transformed into cancer cells under the influence of long-continued irritation.

A similar effect of individual mechanical stimuli can be very well demonstrated in the case of cancer cells. Through such stimuli it is possible to increase the proliferative energy of certain tumors, and such an increase is a cause of the greater virulence of recurrent tumors so frequently observed after operations. But it is also possible to decrease experimentally the proliferative powers of tumors, even in a quantitatively determined way, by exposing tumor cells to the action of physical or chemical means before transplantation.

These and other experiments, which lack of time does not permit to mention, clearly prove how important the study of animal tumors has been and will be still more so in the future for the analysis of cell and tissue growth in general. And, conversely, any progress in our understanding of normal tissue growth will be a step in advance in the understanding of cancer; and we must keep it well in mind that the cancer problem is ultimately a part of the general problem of how the proliferative power of certain cells can be increased apparently indefinitely.

Under certain conditions a long-continued irritation was not required and a single trauma has undoubtedly been followed by the development of a cancer. Even this fact seems now accessible to a rational explanation, since in experiments concerning the nonproduction of the maternal placenta, we learned of the existence of sensitizing substances, specific for certain tissues, and that after a previous sensitization an ordinary trauma may call forth a tumor-like cell proliferation which lasts as long as the organ is sensitized.

The possibility of procuring any desirable number of laboratory animals with growing sarcomas or carcinomas, offered an excellent opportunity for therapeutic experiments.

The results may be summarized as follows: All attempts to influence the growth of tumors by substances which have proved, especially in the hands of Ehrlich, so efficient in the treatment of protozoal diseases, have failed.

Many attempts have been made, especially by Jensen, to prepare, by repeated injection of tumor material into animals of other species, a curative serum. Until recently these efforts did not seem very successful. But quite recently, after a modification of technique, Walker in Liverpool reports much more promising results. In a certain number of cases the serum caused a retrogression or disappearance of the inoculated, growing tumors. The number of his experiments is, however, as yet too small to permit of any definite conclusion.

In the case of the inoculated sarcoma of dogs, Beebe and Crile succeeded in curing animals by the injection of large quantities of the normal blood serum of dogs not immunized. For a full understanding of this result it is necessary to remember that such inoculated sarcomas in dogs are more labile than many other tumors; that they can therefore more easily be induced to retrogress and that in a large number of cases such a retrogression takes place spontaneously.

Positive results were obtained in producing an active immunity against tumor growth. Gaylord and Clowes and also Sticker found that animals, in which a tumor had retrogressed spontaneously, were immune against tumor growth if inoculated a second time. Such a spontaneous retrogression is occasionally observed in transplanted tumors and it can be induced at will, in a large number of cases, by the inoculation of tumor material of experimentally decreased virulence, as my experiments with the sarcoma of rats had proved previously.

Furthermore, Sticker, in the case of dogs, and Ehrlich, Bashford and Schoene, in the case of mice, showed that it is possible to produce an active immunity against a subsequent inoculation with tumor, either by a previous injection of a certain quantity of fresh tumor material, which after transplantation did not grow, or even in the case of the mouse with a suspension of normal liver, spleen or erythrocytes of a mouse.

Before these latter experiments had been undertaken, I had occasion to point out that tumor material of experimentally decreased virulence (e. g. after previous heating) might be of especial use for vaccinating purposes and two years ago Bridre reported successful experiments in which he showed that after exposing tumor material to a moderate heat, an active immunity can be produced in the inoculated animals without much danger of the development of a tumor from the cells which served as a vaccine. This mode of immunization becomes still more important, if we consider that, according to Bashford, only tumors and tissues of the same species can be used for immunization. The presence of an antibody has so far not been proved in actively immunized animals, and it is not certain on what mechanism the active immunity depends.

All these results, important as they are from a theoretic point of view and however promising they may be, at the present time do not admit of any application in man for the following reasons: These immunizing inoculations have so far been effective only if they were made before the tumor had been transplanted; they were without avail, in cases in which the tumor growth had already commenced at the time of the immunization. Furthermore, all these experiments relate almost exclusively to transplanted, not to spontaneous tumors with which alone we have to deal in man. It is quite certain that marked differences exist between the growth of transplanted and of primary tumors, the latter being in all probability much less accessible to the action of therapeutic agencies than the former. It seems to me likely that, if immunization should be attempted in man, it might be best to use the sterile, extirpated tumor of the patient for vaccination, after having decreased its virulence sufficiently through a moderate heating or through similar physical means.

In this brief review, mention must be made of some diagnostic methods which were in part, at least, an outcome of experimental work on animal tumors. R. Weil, in New York, and later Crile and others found that the blood serum, of animals and man affected with cancer, gained certain distinctive hemolytic properties and that, likewise, the erythrocytes behaved in a peculiar manner, and Brieger and his collaborators discovered a change in the antitryptic power of the blood serum in cancer patients. Both of these reactions, but especially Weil's reaction, without being quite specific for cancer, may be used as a supplementary aid in diagnosis.

In conclusion, I wish to state that at the present time the principal weapon in the struggle against cancer still consists in the thorough extirpation of the tumor. In the beginning, cancer is a local disease, although it is not unlikely that certain general conditions exist in the patient which render possible the transformation of normal into cancerous tissue, a transformation that may be so gradual that, by histological examination, it is sometimes impossible to indicate the exact time when such a transformation is beginning to take place. Certain experiences in tumor inoculation prove that the liability to the formation of local metastases may differ even in tumors of the same histological structure. It will therefore be necessary to avoid carefully any contact between the tumor and the surrounding tissues during the operation. Inasmuch as mechanical injury to a tumor may in certain cases lead to an increased energy of growth, it seems to me that, whenever possible, an exploratory incision ought to be followed at once by the radical removal of the tumor. It will be necessary to use all means to secure an early diagnosis and an early operation.

As to preventive measures, the only advice to be given is that long-continued irritation of any kind be avoided as much as possible. But it is only through the complete recognition of the underlying causes, through experimental study, that we can expect to find the rational treatment of cancer. Such experimental investigation, however, requires more means than are usually at the disposal of the investigator and of the university. These means will be forthcoming, if the public realize the importance of this work for its welfare. And the physician, in arousing public interest in these problems, will lay the surest foundation for the discovery of the most efficient treatment.

---

### THE DIFFERENTIAL DIAGNOSIS OF GALLSTONES.

By Christopher Graham, B. S., M. D., Rochester, Minn.

Gall-bladder disease has its peculiar types of digestive disturbances. We would distinguish four stages, based on the degree of symptoms developed in the history. The first is exemplified by those cases of mild disturbance, usually gastric and often lightly considered by the patient, and even more lightly by the physician. These patients have slight attacks of distress with gas and upward pressure, coming often soon after food, or at irregular times, often with sudden onset of short duration and eased by belching or, perhaps, slight vomiting or regurgitation and slipping away almost unnoticed and without treatment, though many and various measures may get credit for what is in reality a natural return to health. These sudden, irregular, mild "dyspeptic" attacks are quite as typical of gall-bladder disturbances as are the severe so-called "typical attacks" which, as a rule, supplant the mild.

The second stage of the disease is seen in those cases with more or less prolonged dull (mild or quite severe) pain in epigastrium, right arch or whole liver area. This pain may be increased by food, exertion or motion. Deep respiration gives pain, and when located entirely posteriorly the trouble may be called pleurisy. These patients pass through prolonged, steady attacks, their distress may alternate with ease, and comparatively good or excellent health may be enjoyed for a time. During an attack, dyspeptic symptoms are prone to be present and but for their irregularity, as compared to ulcer, one might too frequently consider the possibility of a gastric lesion.

In the third class of cases is to be found the greatest number upon whom the correct diagnosis falls, and in this class surgery finds its greatest activity. Here we have the so-called "typical gallstone attack," sudden, severe epigastric pain with radiation to the right arch (at times to the left) through to back or scapular region, spasm of the diaphragm, upward pressure, gas, nausea, vomiting, and, after a longer or shorter terrific attack, comes sudden cessation of symptoms, and, until complications have obtained, an almost immediate return to perfect health. Sudden onset and sudden cessation without apparent cause or any treatment are quite peculiar to gallstone disease when no complications are present. These attacks come irregularly, night and day, and though often called acute indigestion, acute gastritis, gastralgia, neuralgia of the stomach and other equally foreign names, they more often bear no relation to food, and lack all the fundamental symptoms of ulcer.

The fourth condition is that of chronic gall-bladder trouble with adhesions, duct obstruction, perforation, contractions and duct infections with pancreatitis. In this class, chronic gastric disturbances often predominate, and the picture is so closely related to chronic ulcer with complications that a differential diagnosis can not be clearly made, if present symptoms only are considered. At this stage the key to diagnosis depends upon the development of the early history. However, the chief end is obtained when the necessity for surgery is realized, and the patient is sent to the surgeon for relief with a surgical diagnosis of upper abdominal trouble.

Chronic ulcer of the stomach has a definite, clear-cut and regular symptomatology, and we shall discuss this as the purest type of indigestion. Ulcers of the duodenum and pyloric end of the stomach give symptoms that, as a rule, vary in degree only, and often so little that one usually finds greater trouble in differentiation than in making the ulcer diagnosis. However, as the lesion recedes toward the cardia, the clear-cut symptoms lessen and their peculiar pathognomonic character may be lost when the ulcer is clearly away from the pyloric end. First let us note that the histories of those who come to operation cover years of complaint, and that for much of the time the periods of attack and periods of freedom from symptoms alternate. Early in the history the appetite remains good, nutrition does not fail and food brings immediate relief to all symptoms. The pain, distress, gas, sour eructations, nausea and vomiting return one to four hours after meals; the heartier the meal the more marked and prolonged the relief. During the period of attack, this precise relief of symptoms by food or drink and regular return one to four hours later, are peculiarly characteristic and prevail until complications have arisen that seriously interfere with the gastric function. Another stage of the trouble may be considered as beginning after many periods of attack; the peculiar, characteristic symptom-type remains, but is less definite. The attacks are more severe and prolonged, appetite may fail or food is not taken because of pain, distress, gas, vomiting, sour eructations and burning stomach; food and drinks relieve, but the time of relief is shortened and the later pain is increased. Vomiting may replace eructations, and give relief, as does irrigation of the stomach. Nutrition decreases, not so often because of lessened appetite as because of insufficient prescribed diet or fear of late, distressing symptoms. Ease comes for a time from food or drinks, vomiting, irrigation and alkalies, and pain relapses when the acrid contents of the stomach return. It is not the chronicity or the periodicity that is peculiar, nor the degree or location of the pain, neither the vomiting, gas, sour eructations nor sour, burning stomach; these are common to all chronic dyspeptic types of trouble, gallstones, appendicitis, cancer, etc. The characteristic point is the time the symptoms appear, and their regularity after meals; and the equally ready control of symptoms by food, vomiting, irrigation, etc. This regularity and control of symptoms, meal after meal and day after day during the period of attack, is scarcely approached by any other organic or functional disorder. Later when complications have appeared the symptoms change. Food may not give ease, but rather increase the distress, which is often more nearly continuous. There are no periods of real relief. Vomiting occurs perhaps less often, and is more copious and gives partial relief. Appetite and nutrition fail. The early, regular, characteristic history gives us the key to the diagnosis when we are in the presence of the chronic, complicated stages of ulcer development.

The clinical histories of cancer of the stomach seem to fall into three groups: First, those preceded by a clear and prolonged typical ulcer history; second, those in which years before the recent continuous attack, more or less stomach disturbance had been complained of but in which there had been a long period (years) of perfect freedom from symptoms; third, those in which there can be obtained no record of disturbance until the sudden burst of malignant signs. The second group should gain at the expense of the third through careful history taking. At no stage in its development does cancer of the stomach give us a characteristic run of symptoms of its own, as will be found in duodenal and pyloric ulcers and gallstones. The diagnosis is therefore much more difficult to make.

In the first group, which includes about fifty per cent of all cases, there is the long-preceding, typical ulcer history, and this may gradually shade down and become less typical until it approaches the character of complicated ulcer (obstructions, adhesions, perforation, etc.), at which time all typical signs are lost and we have the almost constant symptoms common to complicated ulcer, chronic gallstones, chronic appendicitis, and, in a great measure, those of carcinoma of the stomach also. It is quite impossible to determine when the degeneration begins because the characteristic ulcer symptoms lessen, and gradually assume the complicated type. Appetite may fail, great waste be present, hemorrhage marked, cachexia advanced, free hydrochloric acid absent, and yet ulcer may be the only lesion found at operation. It is in this group of cases that we find the greatest obstacles to an accurate diagnosis. But such late conditions should not be considered medical, and, at the hands of surgeons, the patient should get relief even if the diagnosis be at fault.

In the second and third groups the diagnosis may be oftener and more easily made at the first examination than in the first group, perhaps, because the cancer, developing on the latent ulcer, causes symptoms to appear only when great invasion has taken place, and the patient often presents himself after some lapse of time when the malignant change is so far advanced that little is left to be recommended but palliative remedial measures. In these cases we usually find rapid wasting, cachexia, great loss of strength, a peculiar weakness, loss of appetite, pain (though distress, even mild distress often covers this symptom), tumor, perhaps vomiting with blood and lack of free hydrochloric acid in the stomach contents. The picture is clear and the diagnosis rarely missed. On the other hand, we must be ready to make a tentative diagnosis with one or few signs. Some patients come because of a tumor, some because of weakness and loss of appetite, others only because of wasting and loss of strength. The test meal here reaches its greatest efficiency. (To be continued.)

